# Age as a Risk Factor for Cutaneous Human Anthrax: Evidence from Haiti, 1973–1974

**DOI:** 10.3201/eid0808.020207

**Published:** 2002-08

**Authors:** Arnold F. Kaufmann, Andrew L. Dannenberg

**Affiliations:** Centers for Disease Control and Prevention Atlanta, Georgia, USA

**Keywords:** Bacillus anthracis, cutaneous anthrax, age, Haiti

To the Editor: Few cases of anthrax have been reported in children, in part because most exposures to *Bacillus anthracis* occur in workplace settings. Questions about the susceptibility of children to *B. anthracis* infection were raised when cutaneous anthrax developed in a 7-month-old child in New York City in 2001 after he was taken to visit his mother’s workplace (1). No cases of anthrax were reported in persons <24 years of age in the 1979 inhalational anthrax outbreak in the Soviet city of Sverdlovsk, despite a presumed general population exposure (2). Such reports have led some investigators to postulate that young persons may be less susceptible to anthrax than older persons.

In 1974, the Center for Disease Control reviewed records on the occurrence of human anthrax in District Sanitaire des Cayes, Haiti, as part of an investigation of cutaneous anthrax in a Florida woman exposed to spore-contaminated goatskin drums she purchased in Haiti (3). In 1973, a total of 387 cases (7.6 per 10,000 population) were clinically diagnosed in District Sanitaire des Cayes; another 59 cases occurred in the first 4 months of 1974. All cases were the cutaneous form; gastrointestinal and inhalational anthrax are rarely, if ever, diagnosed in Haiti. The source of infection in these 446 patients could not be determined. Although cases of animal anthrax were rarely reported in Haiti because of a weak surveillance system, 96 (26%) of 368 Haitian goatskin handicraft items were found to be contaminated with *B. anthracis* during the 1974 investigation, suggesting that animal infections were not uncommon (4). Therefore, the source of the infection may have been meat and other products of value salvaged by local residents from anthrax-infected animals.

Age was reported for 366 of the 446 patients in District Sanitaire des Cayes (Table). The distribution of anthrax cases by age group was generally similar to that of the general population, except the proportion of cases in the 15- to 44-year age group was lower than the proportion of persons in that age group in the general population (p<0.03; chi square). In 124 patients for whom information was available, the cutaneous lesion was located on the head or neck (60 patients [48%]), the arm (31 [25%]), the trunk (23 [19%]), and the leg (12 [10%]); this anatomic distribution, reflecting the primary skin contact point of the organism, was similar in all age groups. However, determining whether the various age groups had differences in skin contact exposure leading to infection is difficult. The affected rural population lived in extreme poverty, typically in small huts with dirt floors and no safe water supply or latrine. Malnutrition was epidemic, and nothing edible was discarded. The crowded living conditions limited opportunities to maintain basic personal hygiene and made it likely that exposure to *B. anthracis*–contaminated materials was similar across all age groups. These previously unpublished age-specific anthrax attack rates from Haiti suggest that adults and children have similar susceptibility to cutaneous anthrax.

**Table:** Reported ages of persons with cutaneous anthrax, District Sanitaire des Cayes, Haiti, 1973–74

Age group (yrs)	Anthrax patients No. (%)	General population No. (%)	Cases per 10,000 persons
<1	15 (4.1)	16,361 (3.2)	9.2
1–4	49 (13.4)	59,291 (11.6)	8.3
5–14	96 (26.2)	120,532 (23.5)	8.0
15–44	135 (36.9)	235,164 (45.8)	5.7
45–64	58 (15.8)	64,882 (12.7)	8.9
>65	13 (3.6)	16,669 (3.2)	7.8
All ages	366	512,899	7.1
